# Oxidative Transformations of Lignans

**DOI:** 10.3390/molecules24020300

**Published:** 2019-01-15

**Authors:** Patrik A. Runeberg, Yury Brusentsev, Sabine M. K. Rendon, Patrik C. Eklund

**Affiliations:** Johan Gadolin Process Chemistry Center, Åbo Akademi University, Piispankatu 8, 20500 Turku, Finland; patrik.runeberg@abo.fi (P.A.R.); ybrusent@abo.fi (Y.B.); srendon@abo.fi (S.M.K.R.)

**Keywords:** lignans, oxidation

## Abstract

Numerous oxidative transformations of lignan structures have been reported in the literature. In this paper we present an overview on the current findings in the field. The focus is put on transformations targeting a specific structure, a specific reaction, or an interconversion of the lignan skeleton. Oxidative transformations related to biosynthesis, antioxidant measurements, and total syntheses are mostly excluded. Non-metal mediated as well as metal mediated oxidations are reported, and mechanisms based on hydrogen abstractions, epoxidations, hydroxylations, and radical reactions are discussed for the transformation and interconversion of lignan structures. Enzymatic oxidations, photooxidation, and electrochemical oxidations are also briefly reported.

## 1. Introduction

Lignans constitute a group of natural phenolics found in plant species. They have been identified in around 70 families in the plant kingdom, for example in trees, grasses, grains, and vegetables. Lignans are found in roots, rhizomes, stems, leaves, seeds, and fruits, from where they are usually isolated through extraction with an appropriate solvent. They have a diverse structure built up from two phenyl propane units with different degrees of oxidation in the propane moiety and different substitution patterns in the aromatic rings. Lignans have been shown to have a wide range of biological activities such as antibacterial and insecticidal effects in plants, and anti-cancerous, antiviral, anti-inflammatory, immunosuppressive, anti-diabetic, and antioxidant properties in mammals [1,2,3,4,5,6]. In addition, many lignans have been found in different foods and feeds, and have been associated with health benefits, such as antioxidant activity and anticancer properties [7,8,9,10]. The structure and occurrence of lignans have previously been extensively reviewed in the literature. In some of these reviews the chemical transformations and oxidative degradations have partially been described, mainly for determination of structures [6,11,12,13,14,15,16]. More recently, advances in the chemistry of lignans, including transformations and interconversions of different lignans, were reviewed by Ward [17]. Although synthetic modifications and interconversions have been extensively reported for lignans, a specific overview on oxidative transformations has not been published. Oxidative transformations of lignan structures can be found in various fields: in total synthesis, in biosynthesis, in antioxidant studies, in oxidative degradations, and as part of targeted chemical transformations. In this paper, we report an overview on oxidative transformations of lignan structures, where the focus is put on transformations targeting a specific structure, a specific reaction, or an interconversion of the lignan skeleton. Oxidative transformations related to studies of biosynthetic routes, antioxidant activities, and total syntheses for coupling phenylpropane units (creating the lignan skeleton) are mostly excluded. The purpose of this paper is to exemplify the transformations induced by different reagents or oxidation methods (Table 1). The presented text, figures, and schemes should be taken as representative examples rather than a comprehensive review of the literature. However, the most relevant literature in the field is cited.

## 2. Non-Metal-Mediated Oxidations

### 2.1. 2,3-Dichloro-5,6-dicyano-1,4-benzoquinone (or DDQ)

**Scheme 1:**
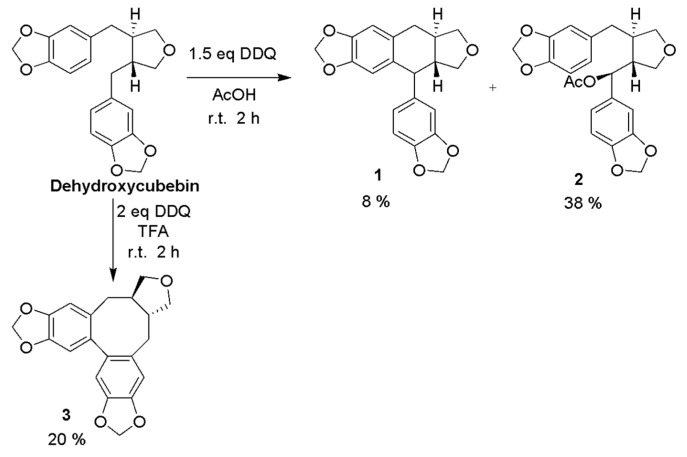
Oxidation of dehydroxycubebin by DDQ. AcOH promotes benzylic functionalization while TFA promotes aryl-aryl coupling.

2,3-Dichloro-5,6-dicyano-1,4-benzoquinone (DDQ) has been used to promote benzylic functionalization of lignans by abstraction of a hydride from the benzylic position. The benzylic cation can then be a target for either intramolecular ring closure or nucleophilic attack. The nucleophilicity of the solvent can direct the reaction towards either benzylic ring closure to aryltetralins, or to aryl-aryl coupling to cyclooctadienes, or toward nucleophilic attack by the solvent. In the latter case, a nucleophilic solvent such as AcOH can function as an oxygen donor. As an example, (−)-dehydroxycubebin was oxidized by DDQ in AcOH, and reacted by either benzylic ring closure to an aryltetralin (**1**), or by *O*-acetylation through nucleophilic attack at the benzylic position (**2**) [18] (Scheme 1). When the solvent was changed to the more acidic and less nucleophilic trifluoroacetic acid (TFA), aryl-aryl coupling to a dibenzocyclooctadiene (**3**) was favored [19,20,21,22,23,24,25,26,27].

**Scheme 2:**
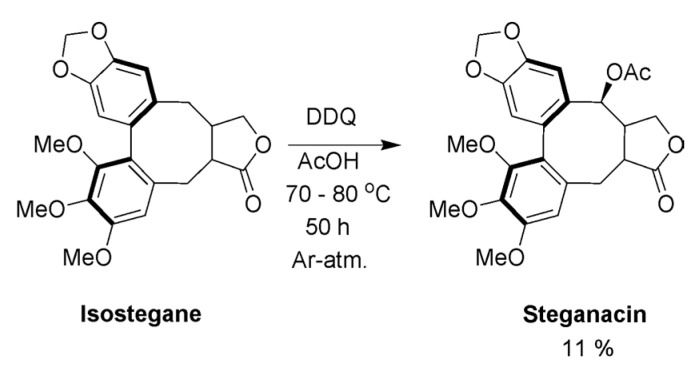
Benzylic *O*-acetylation of (+)-isostegane by 2,3-Dichloro-5,6-dicyano-1,4-benzoquinone (DDQ) in AcOH.

Another example of benzylic *O*-acetylation is shown in Scheme 2. Tomioka et al. reported that the dibenzocyclooctadiene lignan known as (+)-isostegane could be *O*-acetylated at the benzylic position by DDQ in acetic acid, forming (+)-steganacin (Scheme 2) [28].

**Scheme 3:**
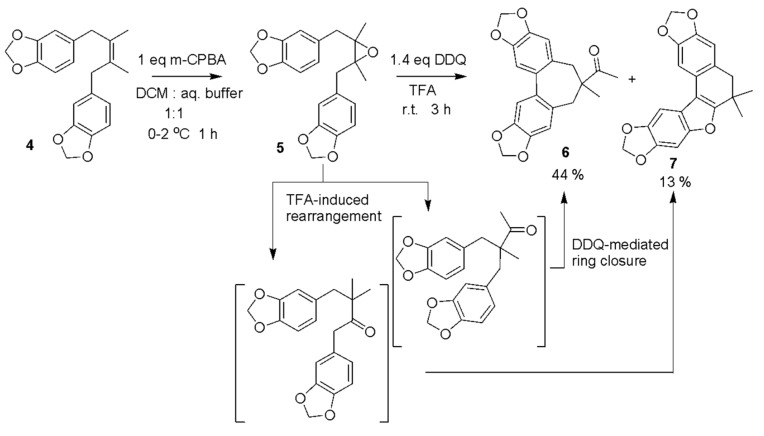
TFA- and DDQ-mediated rearrangement and oxidative ring closure of an epoxide lignan.

A DDQ/TFA-mediated reaction of an epoxy-lignan (**5**), formed through mCPBA epoxidation of an olefinic lignan (**4**), did not form the cyclooctadiene as expected. Instead, TFA-induced epoxide opening and methyl or benzyl group rearrangement to two intermediates preceded DDQ mediated ring closure to **6** and **7** (Scheme 3) [29]. One intermediate underwent the previously described ring closure to a cyclooctadiene, while the other went through benzylic ring closure to a cyclohexanone, with subsequent ring closure to a benzofuran.

**Scheme 4:**
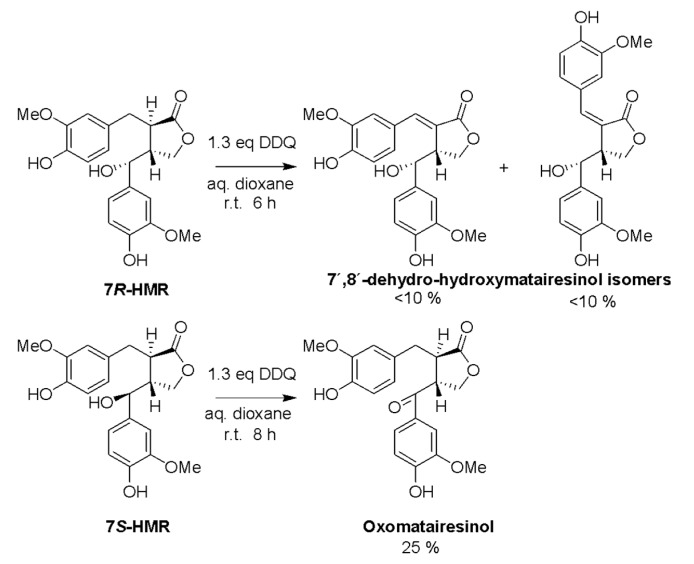
Oxidation of hydroxymatairesinol (HMR) by DDQ is dependent on the stereochemistry of the benzylic alcohol position. The major products are shown here. A range of minor products were also formed in the reactions.

DDQ has also been used for benzylic dehydrogenation of lignan structures to olefins, and in some cases further aromatization to naphthalene type lignans [23,30,31,32]. One example of benzylic olefin formation is visualized in Scheme 4 by the DDQ oxidation of the natural lignan hydroxymatairesinol (HMR) [33,34]. Due to different stereoelectronic properties at the benzylic alcohol for the hydroxymatairesinol epimers, the oxidation reaction resulted in different major products, of which oxomatairesinol had the highest yield. A range of minor products, which are not shown here, were also formed in the reactions. 

**Scheme 5:**
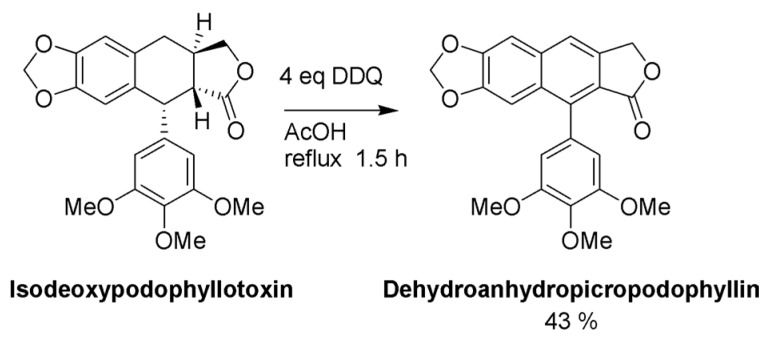
DDQ-mediated aromatization of isodeoxypodophyllotoxin.

An example of DDQ-mediated aromatization of lignans is shown in Scheme 5, for the aromatization of isodeoxypodophyllotoxin [35].

**Scheme 6:**
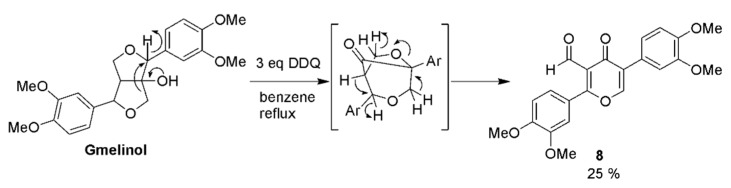
Oxidation of gmelinol with three equivalents DDQ.

When gmelinol reacted with three equivalents of DDQ it underwent benzylic hydride abstraction and C-C-bond rearrangement, followed by an oxidative ring opening of an ether bridge to form an aldehyde, and finally, another benzylic hydride abstraction and formation of olefin (**8**) [36]. The reaction is visualized in Scheme 6.

**Scheme 7:**
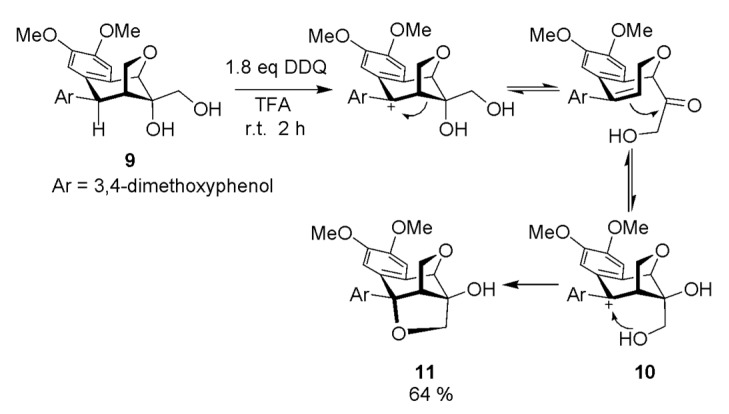
DDQ-mediated epimerization and nucleophilic ring closure.

DDQ-mediated epimerization followed by nucleophilic ring closure at the benzylic position is visualized in Scheme 7 on a lignan (**9**) synthesized from gmelinol [37]. The epimerization step may go through benzylic hydride abstraction, leading to adjacent C-C bond cleavage and regeneration. One epimer (**10**) is favored as it can undergo nucleophilic attack at the positively charged benzylic position, forming product **11**. 

### 2.2. Meta-Chloroperoxybenzoic Acid (mCPBA)

Epoxidation of olefinic lignans with mCPBA has been reported, as previously shown in Scheme 3 [29,38]. 

**Scheme 8:**
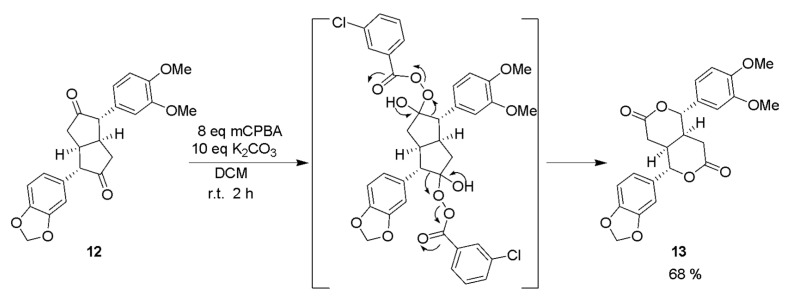
Baeyer-Villiger oxidation of a diketone to a dilactone.

**Scheme 9:**
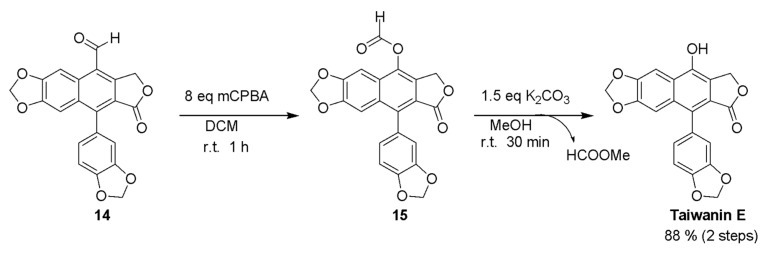
Baeyer-Villiger oxidation of an aldehyde to the corresponding formate, which was further hydrolyzed to the alcohol (taiwanin E).

The Bayer-Villiger oxidation of ketones with mCPBA has also been reported for lignans. The 3,7-dioxobicyclo[3,3,0]octane lignan **12** gave the corresponding dilactone product **13** (Scheme 8) [39,40]. In the total synthesis of taiwanin E, the last two steps consist of Baeyer-Villiger of an aldehyde (**14**) to the corresponding formate (**15**), followed by hydrolysis by MeOH and K_2_CO_3_ to the alcohol (Scheme 9) [41].

**Scheme 10:**
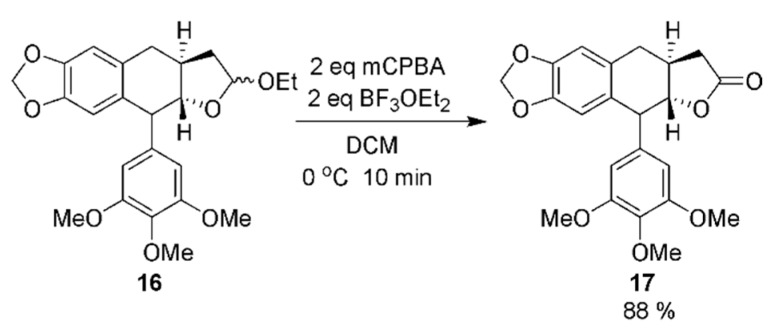
Lewis acid and mCPBA mediated oxidation of an ethoxytetrahydrofuran to the corresponding lactone.

In addition, mCPBA and Lewis acid mediated oxidation of an ethoxy-THF-lignan (**16**) to a lactone (**17**) has been reported (Scheme 10) [42].

### 2.3. Oxidations by Peroxyl Radical

**Scheme 11:**
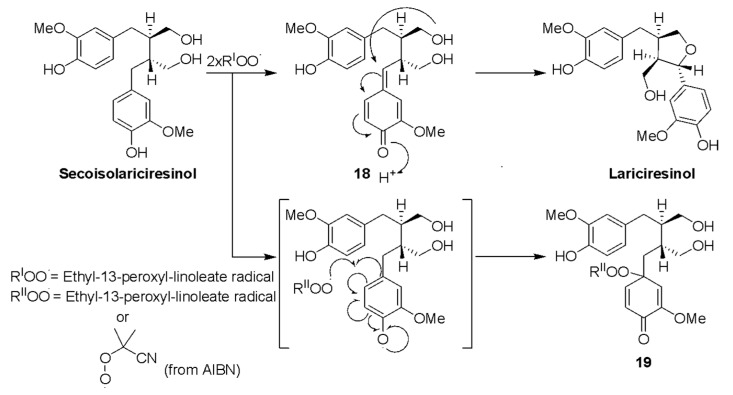
Peroxyl radical mediated oxidation of the lignan secoisolariciresinol.

Radical scavenging of peroxyl radicals has been utilized for measuring antioxidant activity for natural phenolics, including flavonoids [43,44] and lignans [45,46]. The antioxidant activity of lignans is caused by their efficiency to scavenge radicals through hydrogen abstraction, forming phenoxyl radicals. However, in the process, the lignan structure is oxidized and undergoes various transformations. The highly antioxidant active lignan known as secoisolariciresinol was oxidized by two ethyl linoleate peroxide radicals induced by azobisisobutyronitrile (AIBN) to a quinone-like intermediate (**18**), which formed the lignan lariciresinol through trans-selective ring closure. As a side reaction, a peroxyl radical also undertook radical coupling at the *ipso*-position of the phenoxyl radical intermediate to **19** (Scheme 11) [45,46].

### 2.4. Azo Compounds (AAPH, AIBN and ABTS)

2,2′-Azobis(2-amidinopropan) dihydrochloride (AAPH or ABAP), azobisisobutyronitrile (AIBN), and 2,2′-azino-bis(3-ethylbenzothiazoline-6-sulfonic acid) (ABTS) are radical initiators often used in studies of oxidative stability of drugs and proteins, and of antioxidative activity in, among others, food supplements and natural products [47,48,49]. These azo compounds undergo thermal degradation under release of N_2_, and formation of two amidino propane (from AAPH) or isobutyronitrile (from AIBN) radicals. These radicals can be transformed into other reactive radical species depending on the reaction environment, for example by oxygen addition into peroxyl radicals [50].

**Scheme 12:**
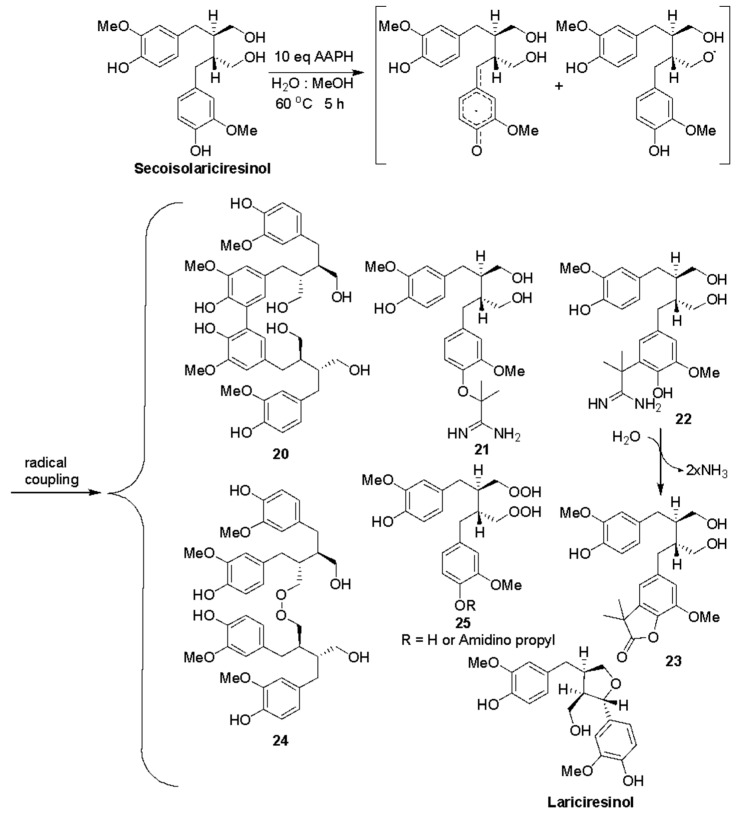
Oxidation products formed from secoisolariciresinol after 2,2′-Azobis(2-amidinopropan) dihydrochloride (AAPH)-mediated radical scavenging.

Studies on antioxidant activity on a range of lignans have been done using AAPH, but only in some cases the major oxidation products have been characterized [51,52,53,54,55,56]. Upon radical scavenging, the lignans form phenoxyl radicals with their radical delocalized over the phenolic ring, followed by radical coupling at either the *ipso*- or *meta*-position, or through the para phenoxyl radical. Alternatively, a second radical reaction yields a quinone methide intermediate that reacts further through an ionic mechanism, as shown in Scheme 11 for **18**. As an example, secoisolariciresinol primarily reacted through an aryl-aryl coupling, the so called 5-5 coupling, to dimer **20**, and through phenoxyl or *meta*-coupling with an amidino propane radical from AAPH to **21** and **22** respectively [57]. Further hydrolysis of the meta-coupled amidino propane unit formed a furanone product (**23**). Lariciresinol was also formed through a similar mechanism as described above (in Scheme 11) for peroxyl radicals. Uniquely for secoisolariciresinol, an alternative radical scavenging path is possible. In this path, an intermediate with alkoxyl radicals on one or both aliphatic hydroxyls, reacts through radical coupling with another hydroxyl radical, forming dimer **24**, or with water, forming hydroperoxides (**25**) (Scheme 12).

As described in the previous chapter, AIBN has been used as a radical initiator for peroxyl radicals in antioxidant studies on lignans [46]. As shown in Scheme 11, a possible antioxidant product is formed through *ipso*-coupling of the lignan with an AIBN derived peroxide. AIBN has also been used in total synthesis of a range of lignans, in aryl halide and Ru_3_SnH/AIBN initiated radical aryl-aryl couplings [58], and in (Me_3_Si)_3_SiH/AIBN promoted radical conversion of a thionocarbonate to a lignan lactone [59]. The radical initiator ABTS has also been used for antioxidant studies on lignans [60,61,62,63], in the so called trolox equivalent antioxidant capacity (TEAC) assays [163]. However, no systematic study on the formed oxidation products by AIBN or ABTS has been done, excluding the product **19**.

### 2.5. 2,2-Diphenyl-1-picrylhydrazyl (DPPH)

**Scheme 13:**
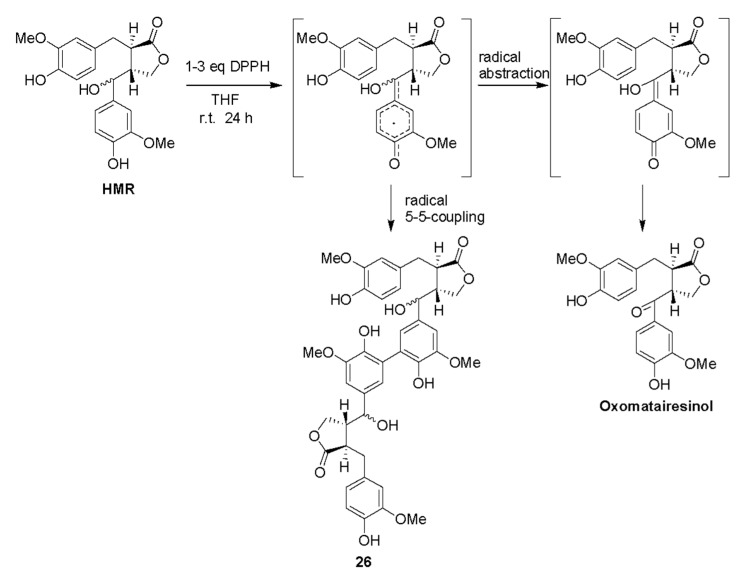
Reaction products of HMR with the free radical DPPH.

DPPH is a stable nitrogen centered free radical that is commonly used for antioxidant assays [64,65]. It has been vastly used for antioxidant assays on lignans [6,66]. Eklund et al. [51] and Smeds et al. [67] investigated the antioxidant mechanism for the DPPH-initiated radical scavenging of different lignans. Upon radical scavenging, the lignan intermediates underwent further radical abstractions or aryl couplings to form dimers and oligomers by 5-5′- or 5-*O*-4′-couplings. An example is shown in Scheme 13 for the reaction between HMR and DPPH. The two major reaction paths, following the initial radical abstraction, were the intramolecular radical coupling forming an aryl-aryl dimer (**26**), or a second radical abstraction and rearrangement to the lignan oxomatairesinol. Further coupling of the dimeric structure also formed larger oligomers.

### 2.6. 2,2,6,6-Tetramethyl-1-piperidinyloxy (TEMPO)

**Scheme 14:**
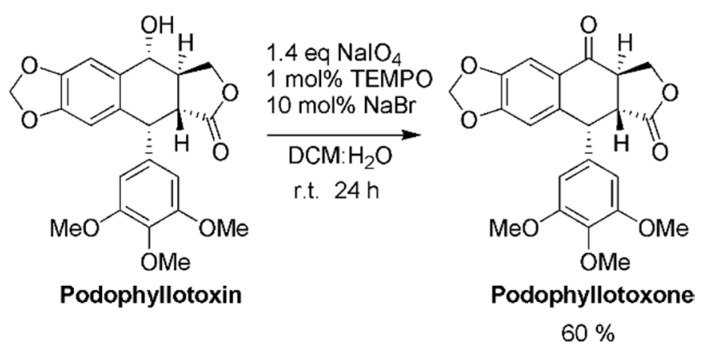
2,2,6,6-Tetramethyl-1-piperidinyloxy (TEMPO)-mediated oxidation of the benzylic alcohol of podophyllotoxin.

The free radical organocatalyst TEMPO has been used in combination with a number of different oxidants. For decades it has been used for oxidation of primary and secondary alcohols [164,165,166]. Surprisingly, TEMPO has not been widely used for the oxidation of lignans. However, a TEMPO-catalyzed oxidation of the benzylic hydroxyl on podophyllotoxin to the corresponding ketone (podophyllotoxone) has been reported (Scheme 14) [68]. Sodium periodate (NaIO_4_) was used as the oxidant in the presence of NaBr as the co-catalyst. 

### 2.7. Hypervalent Iodine Reagents 

The hypervalent iodine reagents BAIB, PIFA, IBX, DMP, and NaIO_4_ have been used in numerous studies for mild oxidation of lignan structures. Efficient oxidation of primary and benzylic alcohols into carbonyls, and phenolics into quinones have been reported. In addition, PIFA has been used for arylic coupling to form benzocyclooctadienes.

#### 2.7.1. [Bis(acetoxy)iodo]benzene (BAIB or PIDA) and [Bis(trifluoroacetoxy)iodo]benzene (PIFA)

**Scheme 15:**
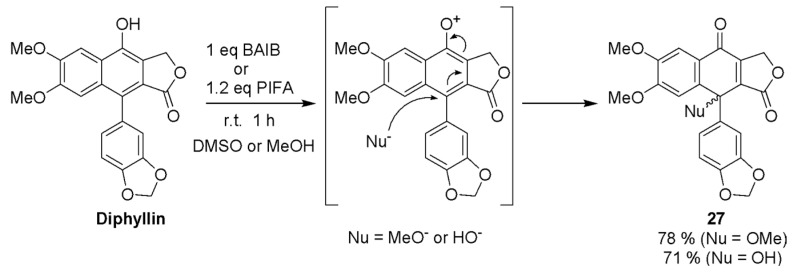
Bis(trifluoroacetoxy)iodo benzene (PIFA) or Bis(acetoxy)iodo benzene (BAIB)-mediated oxidation of diphyllin.

Diphyllin was oxidized by BAIB or PIFA through phenolic hydroxyl oxidation and nucleophilic attack to a para-quinone type product (**27**) (Scheme 15) [69].

**Scheme 16:**
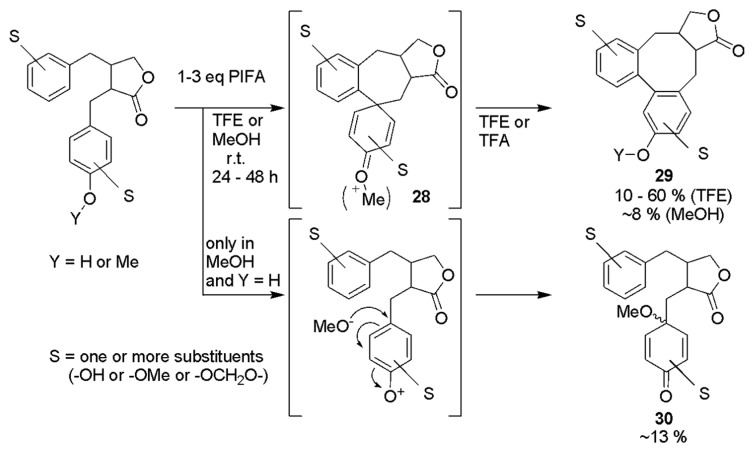
Lignan oxidation by PIFA in TFE or MeOH. With TFE as the solvent, the major reaction was formation of the cyclooctadiene. In methanol, nucleophilic attack occurred as an additional reaction.

Lignans have also been ring closed to cyclooctadienes by PIFA in TFE (Scheme 16, upper reaction path). The initial step is believed to be cyclization to a spirodienone intermediate (**28**) that undergoes rearrangement to the cyclooctadiene product (**29**). In the cases where a lignan has a para-hydroxyl group, in addition to the cyclooctadiene product, nucleophilic addition at ipso-position to **30** was reported when using a more nucleophilic solvent such as methanol (Scheme 16, lower reaction path) [70,71,72,73]. Other minor products were also formed.

**Scheme 17:**
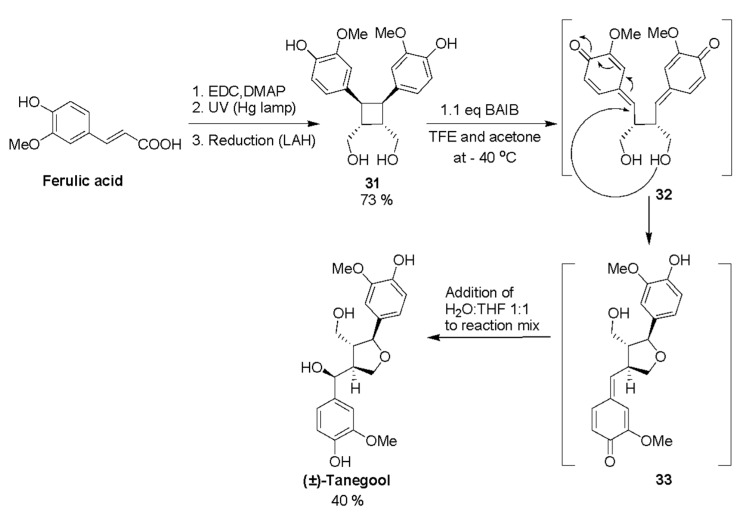
Total synthesis of (±)-tanegool involving BAIB mediated oxidative ring opening.

BAIB has also been utilized in the total synthesis of the natural lignan (±)-tanegool. The synthesis started from ferulic acid. Firstly, a diarylcyclobutanediol intermediate (**31**) was made through a three-step synthesis, involving *para*-nitro-esterification of the carboxylic acid, light induced [2 + 2] coupling, and finally reduction of the *para*-nitro esters with LAH. This intermediate was then oxidatively ring opened to a di-*para*-quinone methide (**32**) by BAIB in TFE and acetone, followed by 5-*exo*-trig cyclization, by one of the hydroxyls, to **33**. The cyclization was stereoselective due to steric hindrance of the bulky aromatic rings. Finally, hydration by addition of water gave (±)-tanegool (Scheme 17) [74]. 

#### 2.7.2. 3-Iodobenzoic Acid (IBX)

**Scheme 18:**
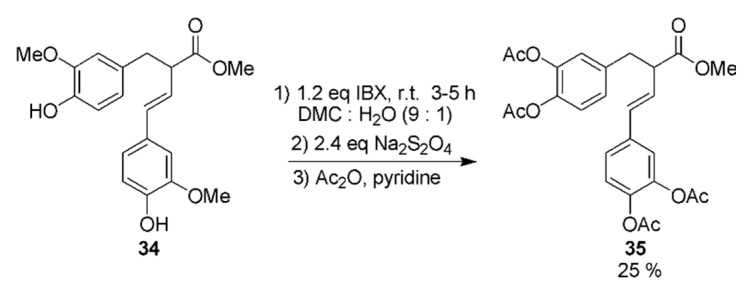
Selective 3-Iodobenzoic Acid (IBX)-mediated demethylation of a norlignan.

A green and selective demethylation reaction, using IBX as the primary oxidant, has been reported for a number of lignans [75]. The substrates underwent oxidation to an *o*-quinone structure, followed by reduction by sodium hydrosulfite to the catechol products. The reaction was selective towards demethylation of phenolic methoxyl groups, leaving methyl esters intact. Scheme 18 shows the IBX oxidation of a methyl ester norlignan (**34**). The reaction mixture was acetylated prior to purification through silica gel column chromatography, giving **35**. The same reaction was also successful on the lignans hydroxymatairesinol (product yield = 55%), conidendrin (product yield = 70%), and lariciresinol (product yield = 40%), yielding demethylated products.

**Scheme 19:**
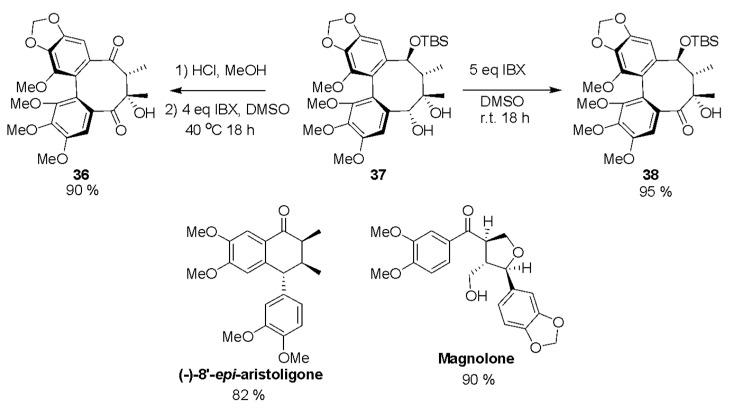
Oxidation of benzylic alcohols by IBX.

When an aliphatic or benzylic alcohol was present, alcohol oxidation to a carbonyl outweighed the demethylation reaction [76]. Scheme 19 shows the selective oxidation of the benzylic alcohol in a synthetic lignan (**37**) to the corresponding ketone (**38**). Alternatively, after removal of the protective group, the product was oxidized to the diketone (**36**). Both of these reactions left the methoxyl groups intact [77]. Benzylic alcohol oxidations by IBX have also been reported on other lignans under similar reaction conditions. Both epi-aristoligone and magnolone have been prepared by this method [78,79] (Scheme 19).

#### 2.7.3. Dess-Martin Periodinane (DMP)

DMP has been reported as an agent for mild and selective oxidation of alcohols to aldehydes and ketones. It has a higher solubility compared to its precursor IBX, and lacks the danger of explosion [80].

**Scheme 20:**
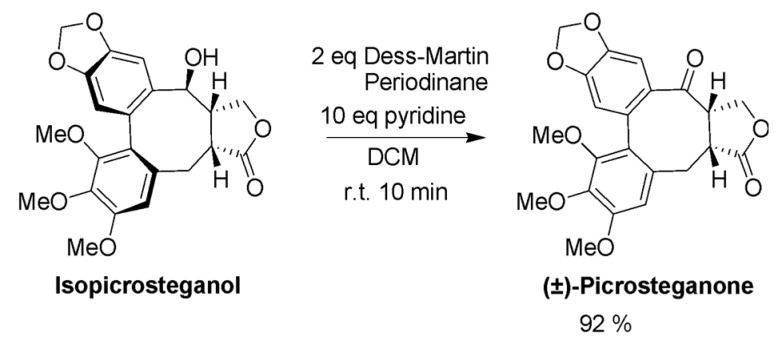
Dess-Martin oxidation of the benzylic alcohol to the ketone.

**Scheme 21:**
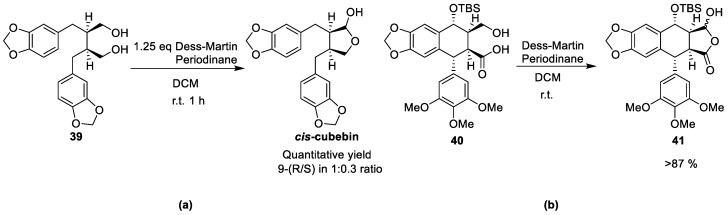
Dess-Martin oxidation of (**a**) diol **39** to cis-cubebin; (**b**) hydroxyacid **40** to hydroxybutyrolactone **41**.

Oxidation of lignans baring benzylic or aliphatic hydroxyls to the corresponding ketones by DMP have been described for various lignans [81,82,83,84,85,86]. An example is shown in Scheme 20 for oxidation of isopicrosteganol to the atropisomers of picrosteganone [87]. Scheme 21a shows the oxidation of an aliphatic primary diol (**39**) with DMP [88]. The reaction quantitatively forms a lactol known as cis-cubebin through subsequent ring closure of the aldehyde. A similar ring closure has been reported by DMP oxidation of a lignan baring a hydroxyl group and a carboxylic acid (**40**), forming a hydroxy-butyrolactone (**41**, Scheme 21b) [89]. 

#### 2.7.4. Sodium Periodate (NaIO_4_)

**Scheme 22:**
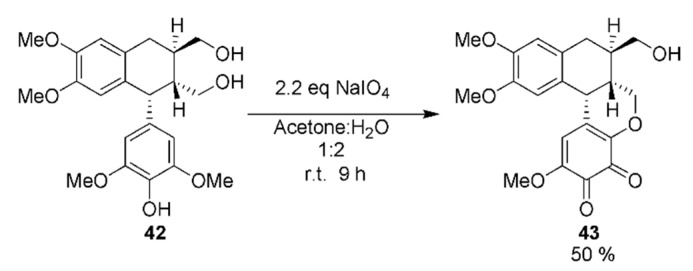
NaIO_4_-mediated oxidation to an ether bridged *o*-quinone structure.

**Scheme 23:**
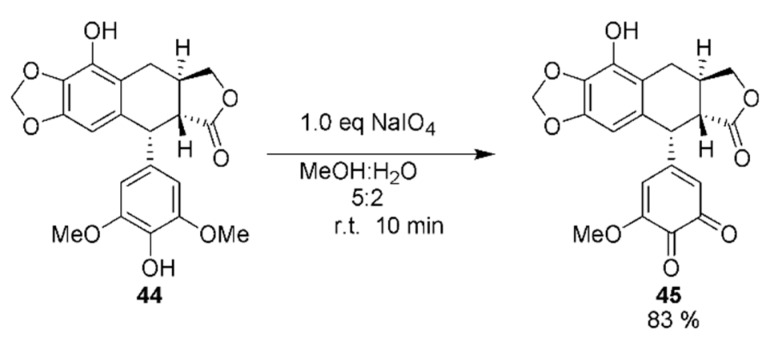
NaIO_4_-mediated oxidation of syringyl-lignan forming an *o*-quinone structure.

In 1955, Adler et al. [90] reported NaIO_4_ mediated oxidation of guaiacyl or syringyl groups, giving demethylation followed by formation of *o*-quinones. When a syringyl lignan (**42**) with aliphatic hydroxyls was oxidized with NaIO_4_, it formed a 5-methoxyl *o*-quinone structure (**43**) with an ether bridge to the ortho-position (Scheme 22) [91]. For lignans without aliphatic hydroxyls, as for **44**, the *o*-quinone structures were formed without the ether bridge (**45**, Scheme 23) [92,93,94].

**Scheme 24:**
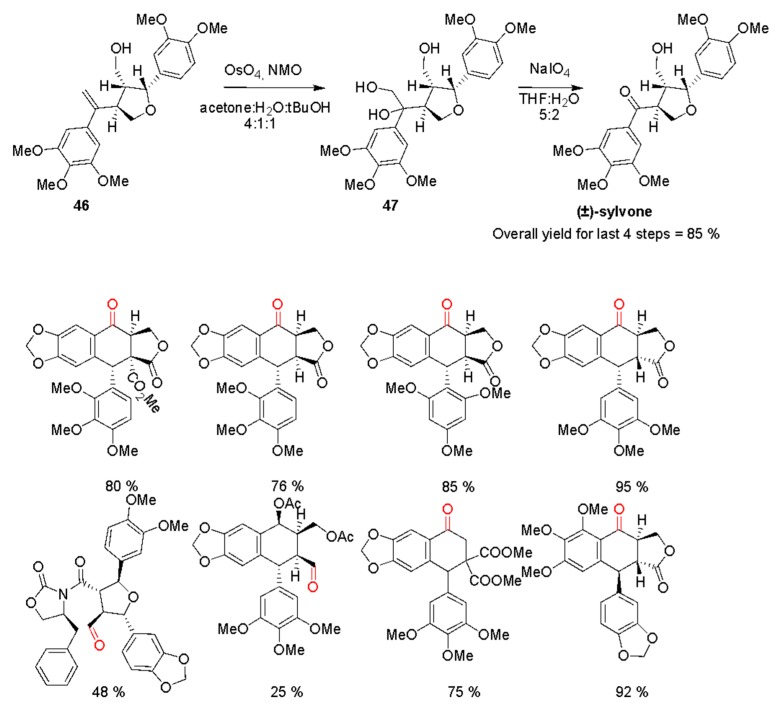
Lemieux-Johnson oxidation in the synthesis of sylvone (**upper**). Other lignan structures where the same methodology has been applied (position for oxidation marked in red). The isolated overall yields for both steps are given (**lower**).

In the total synthesis of sylvone, the final steps consisted of the so-called Lemieux–Johnson oxidation. A vicinal diol (**47**) was formed from an alkene (**46**) by OsO_4_, and then a NaIO_4_-mediated oxidative cleavage of the vicinal diol gave the ketone (sylvone) and formaldehyde (Scheme 24) [95]. Lemieux-Johnson oxidation has been used on other lignans as well, giving ketone products in high yields, as shown in Scheme 24 [96,97,98,99,100]. 

### 2.8. N-Bromosuccinimide (NBS)

**Scheme 25:**
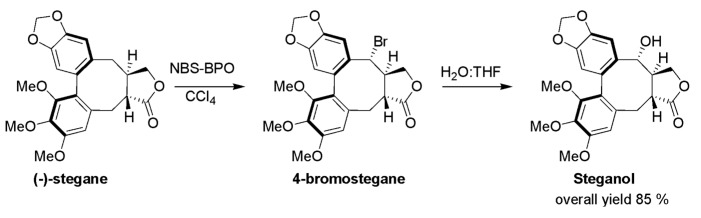
Wohl-Ziegler bromination of (+)-isostegane followed by hydrolysis to (−)-steganol.

The Wohl-Ziegler reaction using NBS and a radical initiator to promote arylic or benzylic bromination has been widely used, and also applied to lignans. Tomioka et al. reported a benzylic bromination of (−)-stegane by NBS in CCl_4_, using benzoyl peroxide (BPO) as the radical initiator [101]. The formed 4-bromostegane yielded (−)-steganone after hydrolysis in aqueous THF, with an overall yield of 85% (Scheme 25). By further acetylation, the desired product (−)-steganacin was obtained in 72% yield. Similar results were also achieved starting from stegane, a stereoisomer of (+)-isostegane [35].

**Scheme 26:**
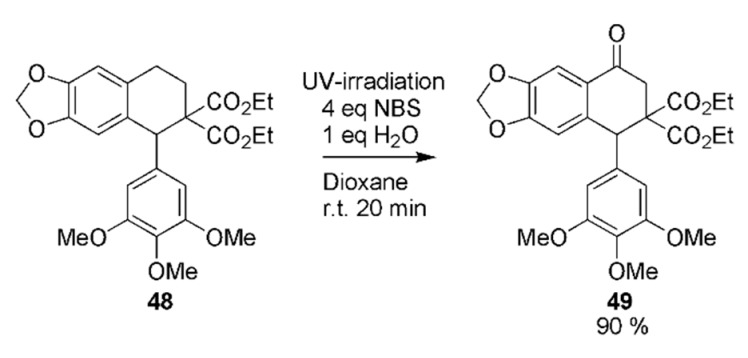
UV and NBS-mediated formation of the benzylic ketone (**49**).

By using a solvent with increased polarity, NBS can also be used to oxidize alcohols to aldehydes and ketones [102]. This has been imposed on a photolytic NBS-mediated oxidation of lignan **48**, using dioxane as the solvent, forming a benzylic ketone (**49**) product in high yield (Scheme 26) [103]. The reaction was proposed to proceed through bromination of the benzylic position, followed by hydrolysis by water to a hydroxyl, and finally oxidation to the corresponding ketone. 

**Scheme 27:**
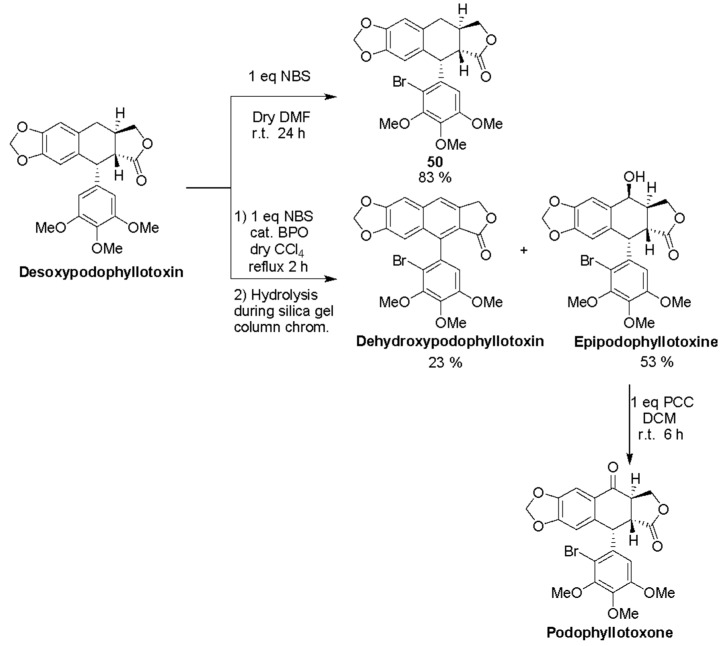
Reaction of deoxypodophyllotoxin with NBS in DMF and CCl_4_, and further oxidation of epipodophyllotoxine by PCC to the corresponding ketone (podophyllotoxone).

When a similar method was applied to deoxypodophyllotoxin by a reaction with NBS in DMF, only arylic bromination occurred (**50**, Scheme 27) [104]. To get the benzylic ketone, NBS-BPO oxidation in CCl_4_ followed by hydrolysis was employed. The product epipodophyllotoxine was further oxidized by pyridinium chlorochromate (PCC) to podophyllotoxone. Interestingly, in addition to epipodophyllotoxine, the NBS-mediated reaction also yielded dehydroxypodophyllotoxin through oxidative aromatization [105]. 

**Scheme 28:**
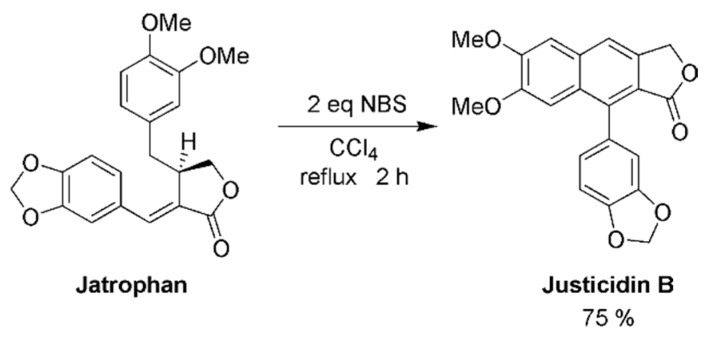
Synthesis of justicidin B by NBS-oxidation of jetrophan.

A similar naphthalene type lignan, known as justicidin B, was formed through NBS-treatment of jatrophan in CCl_4_ (Scheme 28) [106,107]. The reaction includes trans-cis-isomerization, oxidative ring closure, and dehydrogenation. 

### 2.9. Dimethyldioxirane (DMDO or DMD)

**Scheme 29:**
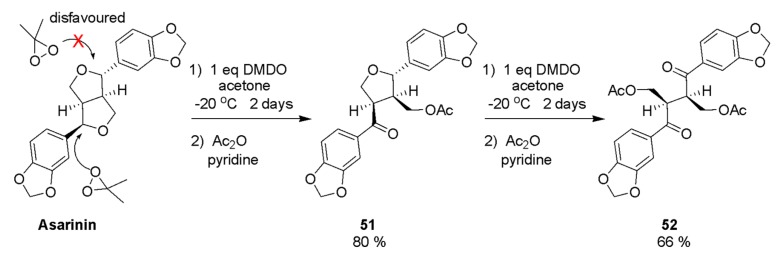
Selective ring opening of asarinin by DMDO.

A highly selective ring opening of asarinin to a diastereomerically pure product (**52**) when using DMDO has been reported (Scheme 29) [108]. With one equivalent of DMDO, a selective ring opening was achieved at the C-7 with *R* configuration, while the other furan ring remained unaltered (**51**). DMDO was highly reactive towards the substrate, and the reaction needed to be performed at −20 °C. If more than one equivalent of DMDO was used, the reaction was believed to go through a radical mechanism yielding a mixture of products. However, after acetylation of the product, ring opening of the second furan ring was achieved under the same conditions as previously used, only with a slightly lower yield.

### 2.10. Nitrobenzene

Reports from the 1950s and 1960s show oxidative degradation of lignans to vanillin, vanillic acid, and syringaldehyde, using nitrobenzene as the oxidant. Nitrobenzene oxidation of thujaplicatin and dihydroxythujaplicatin, which are lignans found in western red cedar, gave vanillin as the sole product, but only in approximately 2% yield [109,110]. Thujaplicatin methyl ether, with the *meta*-hydroxyl protected as a methoxyl group, gave around 4% of both vanillin and syringaldehyde upon nitrobenzene oxidation. Another study systematically oxidized a range of lignans under the following conditions: 180 °C, 60 ml of 2 M NaOH and 8 mL nitrobenzene per gram of lignan. The reaction time was 2 h. The results are listed in Table 2 [111]. Similarly, nitrobenzene has also been used for the production of vanillin from industrial lignin [167]. 

## 3. Metal-Mediated Oxidations

Oxidation of lignans using metal reagents could be divided into methods which use equimolar amounts or catalytic amounts of the metal oxidant. 

Catalytic reactions usually employ transition metals in heterogenous or homogenous conditions and may be sensitive for different functional groups or heteroatoms. Equimolar metal oxidants are widely used for all types of oxidation of lignans, and may involve one-electron oxidations or ionic mechanisms. 

### 3.1. Chromium (VI) Oxidations

**Scheme 30:**
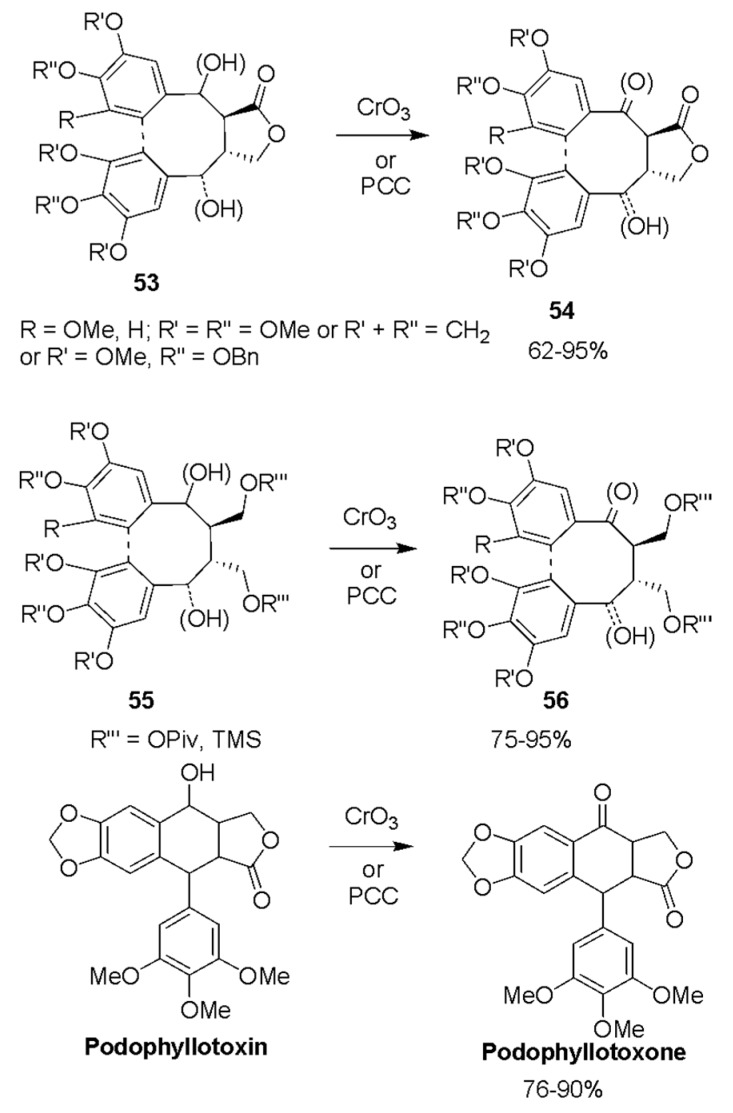
Oxidation of the benzylic alcohol to the corresponding ketone by Cr(VI) oxidants. The dotted line corresponds to either the existence of the bond or the absence of the bond.

There are many examples of Chromium (VI) oxidation of benzylic alcohols to ketones in different lignan structures. Acyclic matairesinol derivatives, [112,113,114], cyclooctadiene structures (**53**–**56**) [87,115], and podophyllotoxin derivatives have all been oxidized by CrO_3_ or PCC to the corresponding ketone in relatively high yields (60–95%) [103,105,116,117] (Scheme 30).

**Scheme 31:**
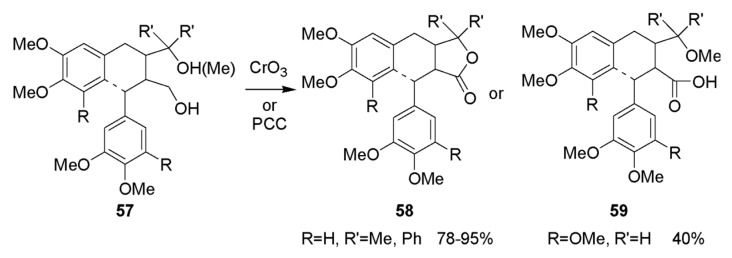
Oxidation of the primary alcohols by Cr(VI) oxidants. The dotted line corresponds to either the existence of the bond or the absence of the bond.

Cr(VI) mediated oxidation of primary alcohols in the lignan structures (**57**) has also been performed. Oxidation by PCC or CrO_3_ led to lactones and carboxylic acids (**58** and **59**) [118,119,120] (Scheme 31).

There are no literature examples where chromium (VI) oxidants have successfully been used for oxidation of lignans in the presence of free phenolic groups. Therefore, it can be concluded that chromium mediated oxidations cannot be performed when phenolic groups are present in the structure.

### 3.2. Palladium and Gold Mediated Oxidations

**Scheme 32:**
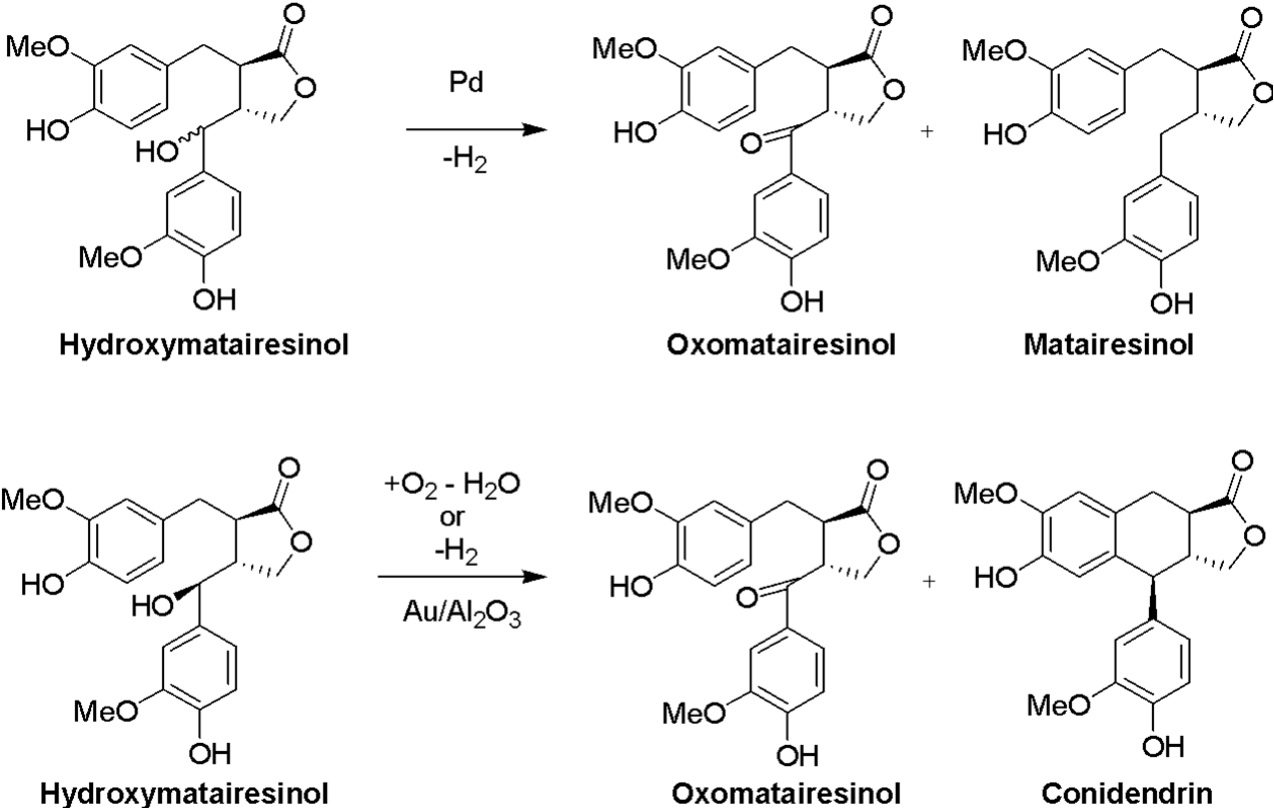
Palladium and gold catalyzed oxidation (dehydrogenation) of hydroxymatairesinol.

Oxidation of benzylic alcohols in the presence of free phenolic groups has been achieved by transition metal catalysis. Hydroxymatairesinol (HMR) has been shown to undergo catalytic dehydrogenation in mild conditions, using palladium on different supporting materials [121] (Scheme 32). The major oxidation product was oxomatairesinol, however, hydrogen formed during the dehydrogenation partially reacted with HMR by hydrogenolysis to give a significant amount of matairesinol as a side product. Later on, it was shown that the same transformation could also be performed using gold as a catalyst [121]. This method was much more selective towards oxomatairesinol [123] and showed faster conversion in the presence of oxygen [124,125,126]. It is noteworthy that one diastereomer of HMR gave oxomatairesinol much more selectively and with higher conversion rates, but some conidendrin was also formed as a side product depending on the conditions of the reaction.

### 3.3. Molybdenium Mediated Oxidations

**Scheme 33:**
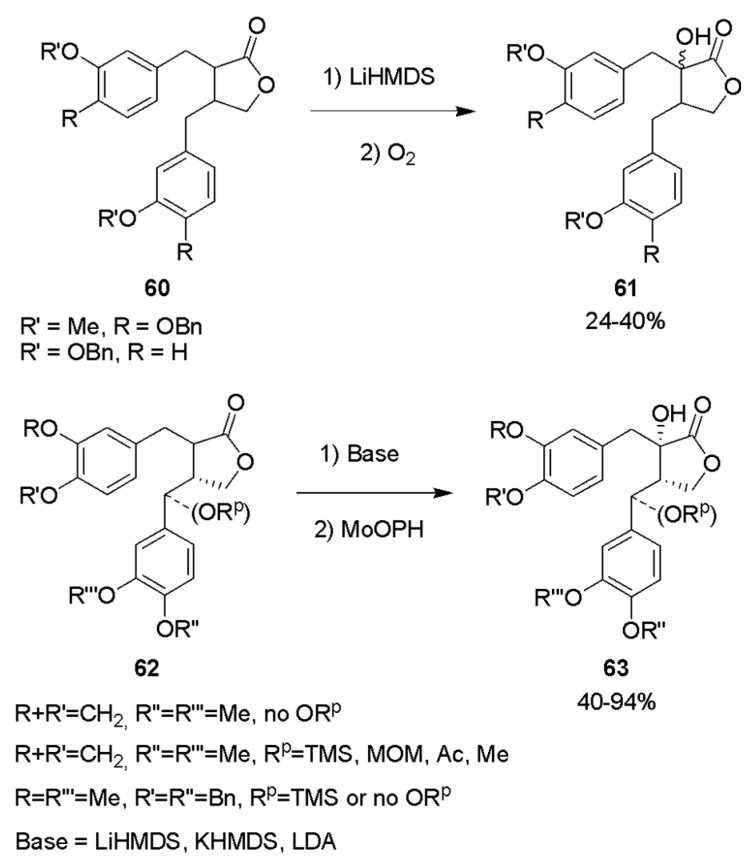
Molybdenum catalyzed α-hydroxylation of butyrolactone lignans. The dotted line corresponds to either the existence of the bond or the absence of the bond.

Hydroxylation at the α-position of butyrolactone lignans (**60**) has been performed directly on the deprotonated ester by molecular oxygen [127,128]. However, this reaction proceeded much faster and with better selectivity when molybdenum reagents were used. For this purpose, oxodiperoxymolybdenum (pyridine) (hexamethylphosphoric triamide), MoO5·Py·HMPA(MoOPH), also known as Vedejs’ reagent was used [113,129,130,131]. The stereoselectivity of the oxidation was very much dependent on the base and the conditions used for the deprotonation. For example, if KHMDS was used, the diastereomeric excess of one isomer was just 11% [113,130]. At the same time, when KHMDS was used together with 18-crown-6, the selectivity raised to 64–99% [130,131] (Scheme 33).

**Scheme 34:**
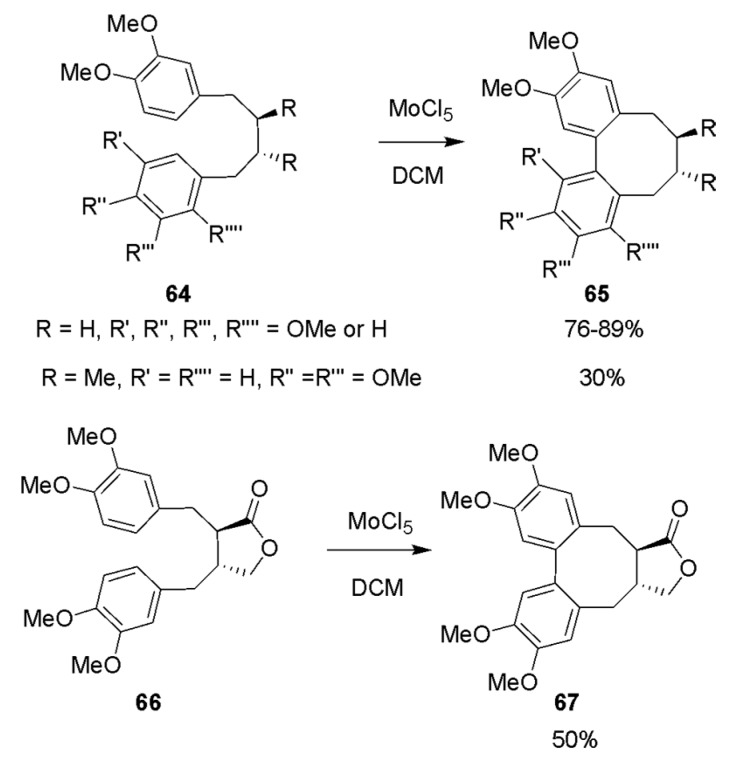
Ar-Ar oxidative coupling by MoCl_5_.

Molybdenum reagents have also been used for the preparation of 2-2′-cyclolignan structures. Oxidative coupling of two aryls was performed with molybdenum pentachloride (MoCl_5_) [132,133]. The formation of the cyclooctadiene structure proceeded with excellent yields when the substrates were non-functionalized aliphatics, but the yields decreased dramatically with increasing degree of functionalization (Scheme 34).

### 3.4. Vanadium, Thallium and Ruthenium Oxidations

**Scheme 35:**
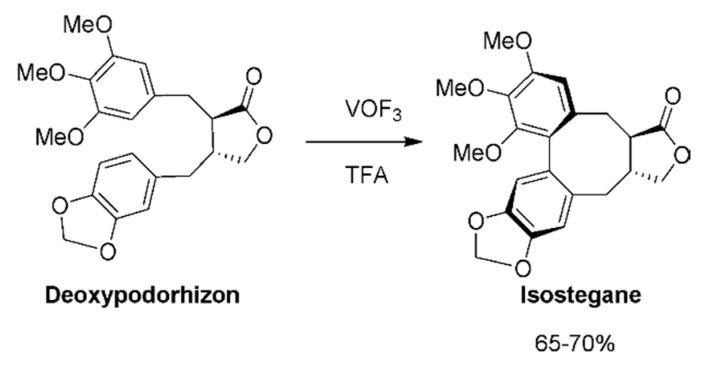
Ar-Ar oxidative coupling by VOF_3_.

Oxidative coupling has also been reported using V, Tl, and Ru. Vanadinum oxofluoride in the presence of trifluoroacetic acid has shown good selectivity for the Ar-Ar oxidative coupling, forming the cyclooctadiene lignan structure Isostegnane in high yield [134] (Scheme 35).

**Scheme 36:**
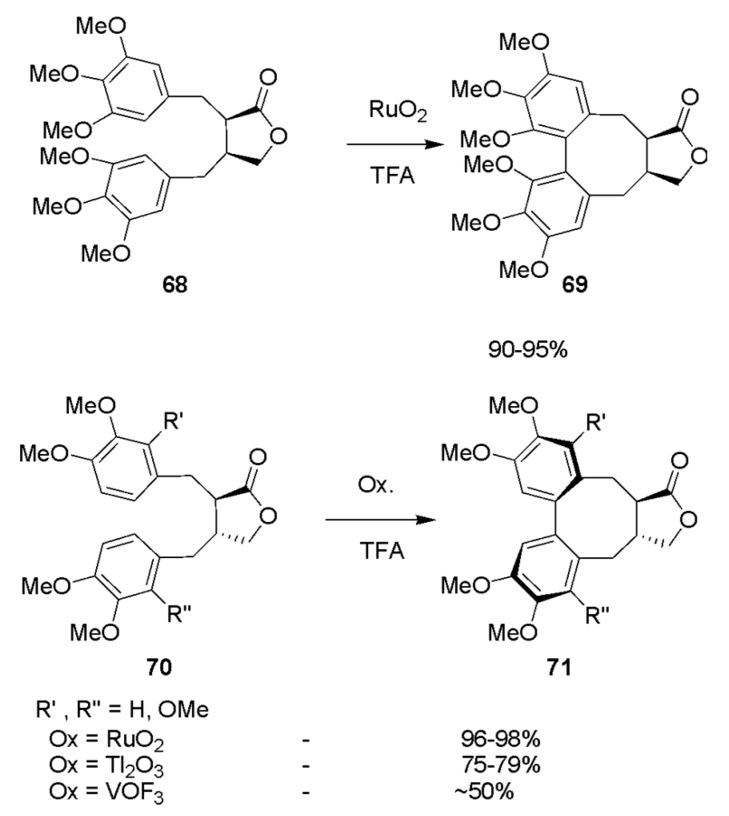
Ar-Ar oxidative coupling by VOF_3_, RuO_2_, or Tl_2_O_3_.

Later it was also shown that thallium oxide (Tl_2_O_3_) or ruthenium oxide (RuO_2_) also work well as oxidants in this reaction [135,136]. In fact, the reactions with ruthenium oxide showed higher yields than reactions with thallium oxide, and vanadium oxofluoride reactions showed slightly lower yields in the vast majority of the reported reactions [137,138,139,140,141]. High stereoselectivity was observed in most cases (Scheme 36).

**Scheme 37:**
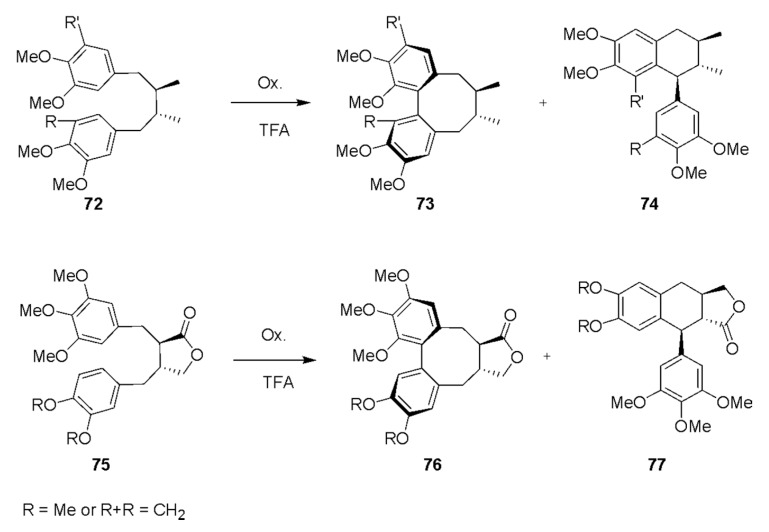
Ru, Tl, and V mediated oxidative cyclizations, forming 2-2′ and 2-7′cyclolignans.

In many of these reactions, 2-7′-cyclolignans (**74**) and (**77**) were formed as minor products by oxidation of the benzylic positions [140,142,143,144] (Scheme 37).

**Scheme 38:**
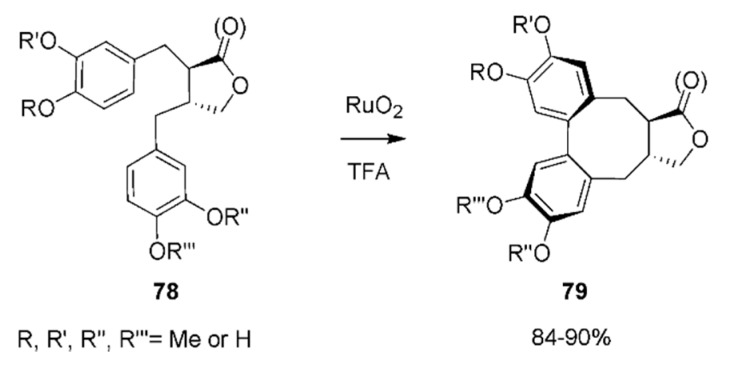
Oxidative coupling of matairesinol derivatives in the presence of free phenolic groups.

Interestingly, these reactions also proceeded in the presence of a free phenolic group. Yields up to 90% of **79** were obtained with matairesinol derivatives (**78**) [137,144,145,146] (Scheme 38).

It has also been reported by Planchenault et al. [145,147] that some metal oxidants other than Mo, V, Ru, and Tl, such as Mn(OAc)_3_, Ce(OH)_4_, Re_2_O_7_, Fe(OH)(OAc)_2_, Co_3_O_4_, Ag(OCOCF_3_)_2_, CrO_3_, IrO_2_, Pr_6_O_11_, SeO_2_, TeO_2_ etc., are possible to use for high yield preparations of cyclooctadiene-lignans.

### 3.5. Methyl Trioxo-Rhenium (MTO) Catalyzed Oxidations

**Scheme 39:**
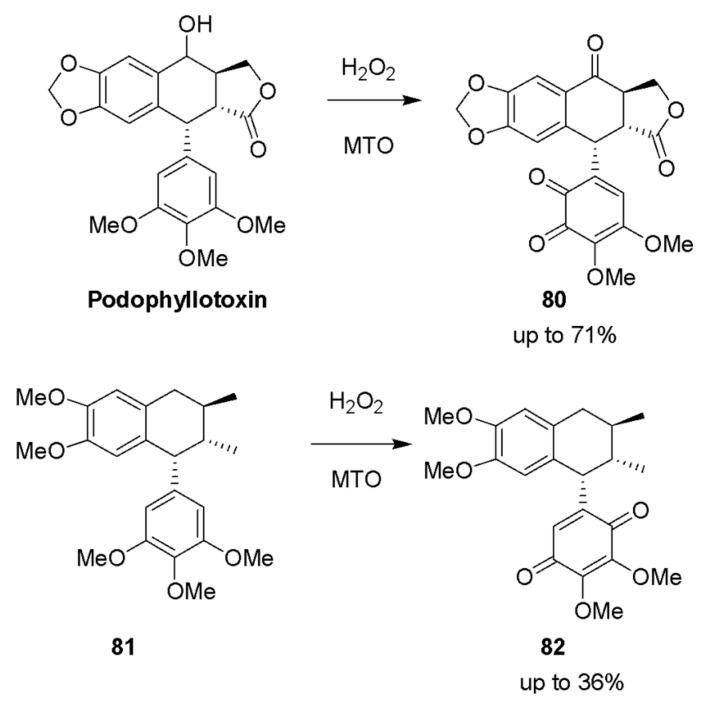
Oxidation of podophyllotoxin and related structures by Methyl Trioxo-Rhenium (MTO).

MTO catalyzed oxidations are interesting because of the diversity of possible transformations. For example, podophyllotoxin and related structures (**81**) were oxidized to quinone structures (**80** and **82**) by MTO [148] (Scheme 39). The reaction proceeded via demethylation, hydroxylations, and at the same time, the benzylic alcohol was oxidized.

**Scheme 40:**
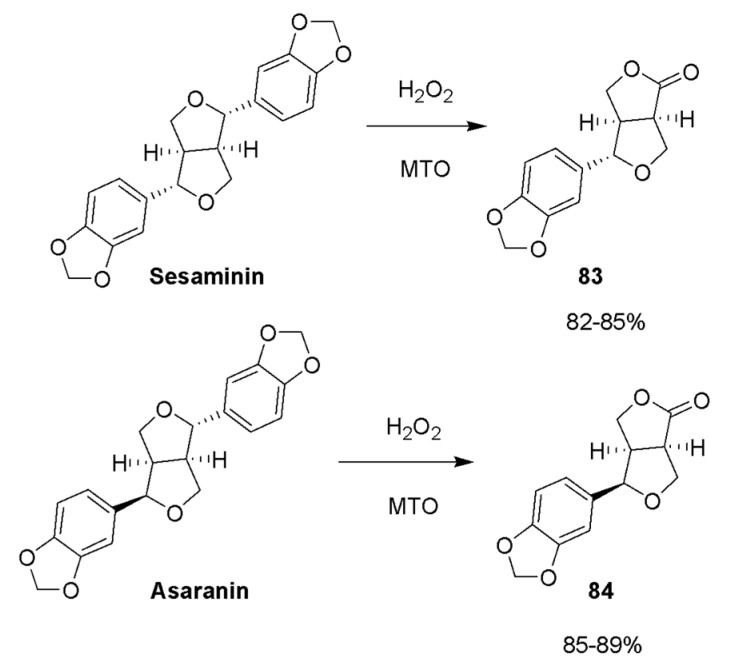
Oxidation of asaranin and sesaminin with MTO.

When asaranin and sesaminin were treated with MTO in similar conditions, the reaction resulted in cleavage at the benzylic position to yield lactones (**83** and **84**) [149]. (Scheme 40).

**Scheme 41:**
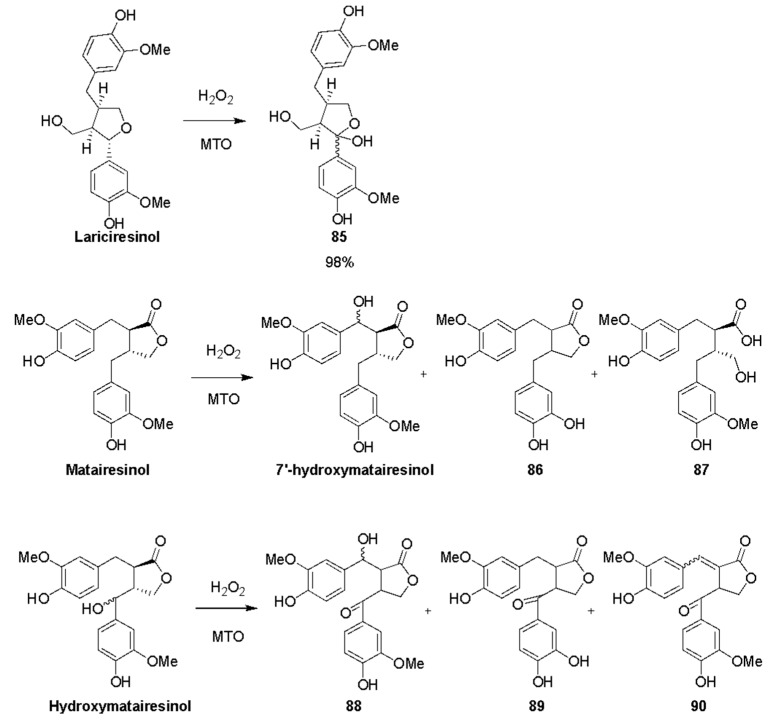
Oxidation of lariciresinol, matairesinol, and hydroxymatairesinol by MTO (major products shown).

MTO catalyzed reactions with lariciresinol, matairesinol and hydroxymatairesinol resulted in multiple benzylic hydroxylations, demethylations, oxidations, and cleavage of water [150] (Scheme 41, structures **85**–**90**).

### 3.6. Other Metal Mediated Lignan Oxidations

**Scheme 42:**
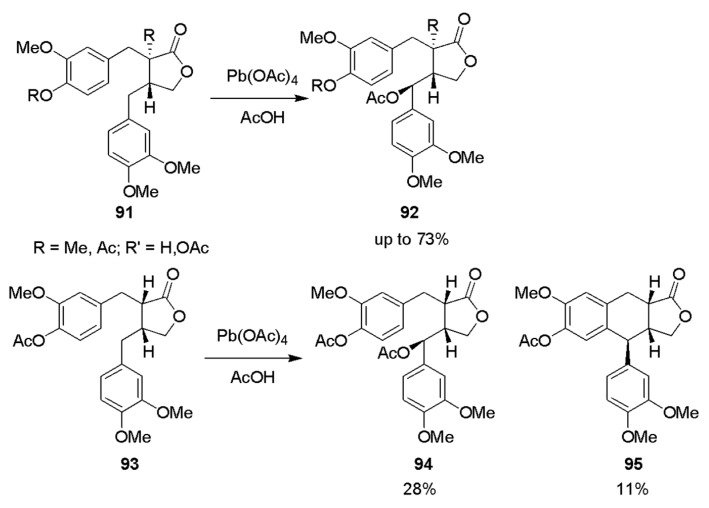
Lead acetate acetoxylation of matairesinol derivatives.

Oxidative acetoxylation of matairesinol derivatives **91** and **93** has been accomplished using lead acetate [151] (Scheme 42). In the reactions of the *trans*-butyrolactone derivatives (**91**), one diastereomer of the 7-acetoxy product (**92**) was formed in high yield (60–73%). In the reaction of the cis lactone (**93**) in the same conditions, the reaction resulted in a mixture of the 7-acetoxy product (**94**) and the 2′-7-cyclolignan (**95**).

**Scheme 43:**
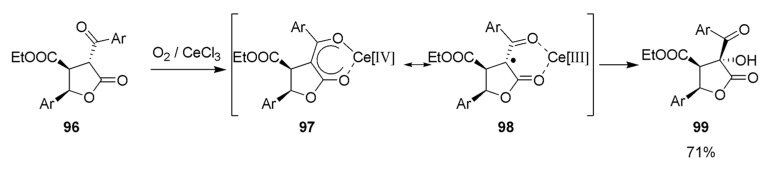
Cerium trichloride mediated α-hydroxylation.

In addition to molybdenum initiated α-hydroxylation of esters, cerium trichloride catalyzed α-hydroxylation has also been reported [152]. This method did not require deprotonation of the α-position. Coordination of cerium to two α-carbonyls directed the position of the oxidation. (Scheme 43).

## 4. Other Oxidation Methods

### 4.1. Enzymatic Oxidations

Although enzymatic oxidations have mostly been studied for elucidation of biosynthetic routes for many lignans, some attempts for targeted oxidations in vitro have been reported. 

Laccases are copper-containing oxidases which have been used in, among others, wood, pulp, and paper industries [168]. They play a key role in the dimerization of 4-hydroxycinnamic acids in the biosyntheses of natural lignans [22,169]. Lignans have also been used as substrates in laccase activity studies [170,171]. Laccase oxidations of lignans baring guaiacyl groups have led to efficient polymerization [172,173,174]. The mechanism is believed to involve hydrogen atom abstraction to form phenoxyl radicals, which then undergo intermolecular radical couplings [175]. 

**Scheme 44:**
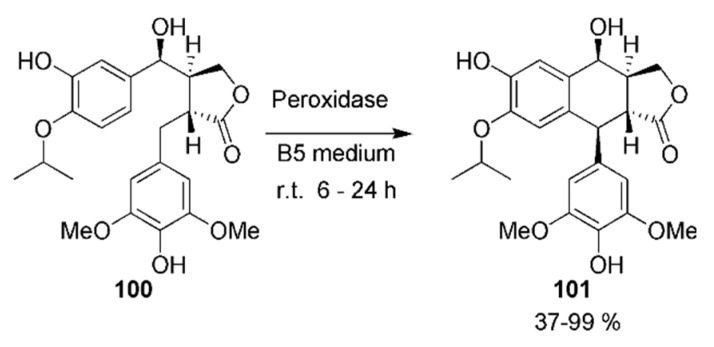
Enzymatic, peroxidase-mediated ring closure.

Peroxidases are another group of oxidases which are also involved in the biosynthesis of lignans through β-β coupling of two phenylpropanoid units [22,169,176,177]. They typically use hydrogen peroxide as cofactor in the oxidation reactions. A range of different peroxidases has been used for selective ring closure of butyrolactone lignans (ex. **100**) with up to quantitative yields of the aryltetralin product (ex. **101**) (Scheme 44). The reactions were performed with both immobilized cell cultures and freely suspended plant cell cultures, and in absence of foreign hydrogen peroxide. Although the reaction worked also in hexane, quantitative ring closure only occurred when the reaction was done in B5 medium [153,154]. A similar reaction, but with H_2_O_2_ as a cofactor and ethanol as solvent, has also been reported on the same lignan [155]. 

**Scheme 45:**
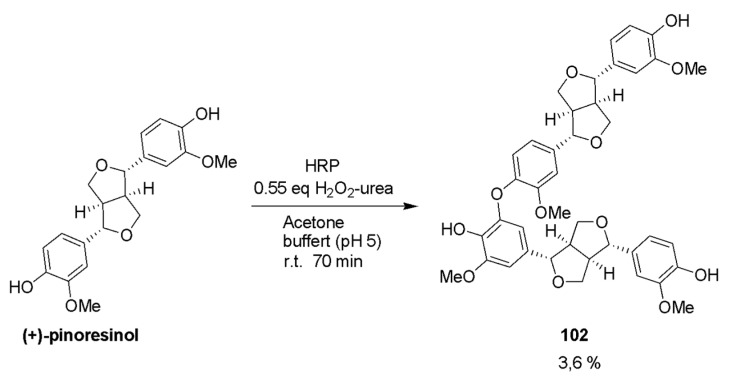
Peroxidase and H_2_O_2_- mediated 4-*O*-5-coupling.

A study of 4-*O*-5 units in softwood lignin used pinoresinol, among others, as a lignin model compound. A pinoresinol dimer (**102**) coupled through a 4-*O*-5 bond, was synthesized using horseradish peroxidase (HRP) and a hydrogen peroxide-urea complex (Scheme 45) [156]. Although sufficient for the study, the isolated yield was very low.

**Scheme 46:**
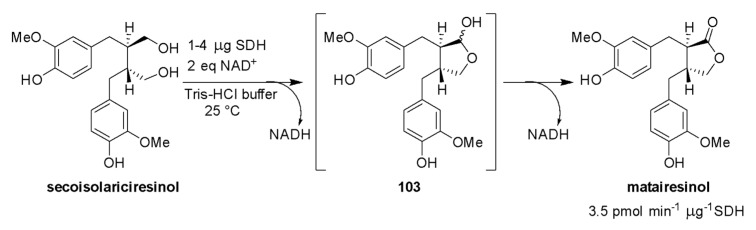
Enzymatic transformation of secolariciresinol into matairesinol.

In the biosynthesis of matairesinol, secoisolariciresinol is oxidized by an enzyme known as secoisolariciresinol dehydrogenase (SDH) [178,179]. The reaction proceeds through an oxidative ring closure of the diol to a lactol, and further oxidation to the lactone. In a study on the mode of catalysis of SDH, the reaction was done in vitro, in a 5 mM scale, using isolated SDH and NAD^+^ in a Tris-HCl buffer (Scheme 46) [157].

**Scheme 47:**
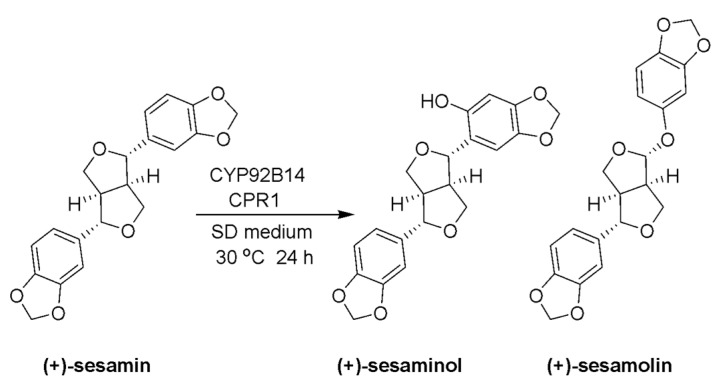
Enzymatic transformation of sesamin into sesaminol and sesamolin.

Enzymatic oxidation of sesamin has been studied in vitro, using yeast cells expressing some of the enzymes found in sesame seeds [158]. Yeast strains containing a combination of a P450 enzyme (CYP92B14) and *Sesamum indicum* cytochrome P450 oxidoreductase 1 (CPR1) in a synthetic defined (SD) medium gave, via oxidation and rearrangement, sesaminol and sesamolin (Scheme 47), two lignans found in sesame seeds.

**Scheme 48:**
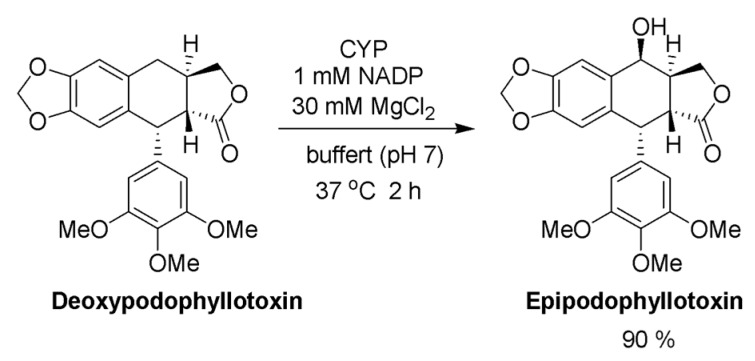
Enzymatic bioconversion of deoxypodophyllotoxin into epipodophyllotoxin.

*Escherichia coli* expressing human hepatic enzymes (CYP) have been used for the bioconversion of deoxypodophyllotoxin into epipodophyllotoxin (Scheme 48) [159]. The objective of the study was to investigate a possible route for industrial production of epipodophyllotoxin, which is a valuable precursor for the pharmaceutical industry. High conversions were achieved, but the reactions were done in scales up to only 0.5 μmols. The high cost of up scaling was presented as the bottleneck that needed to be tackled before a possible industrial production. A large scale isolation of deoxypodophyllotoxin, from wild chervil (*Anthriscus sylvestris*), had already been accomplished [180]. 

### 4.2. Electrochemical Oxidations

**Scheme 49:**
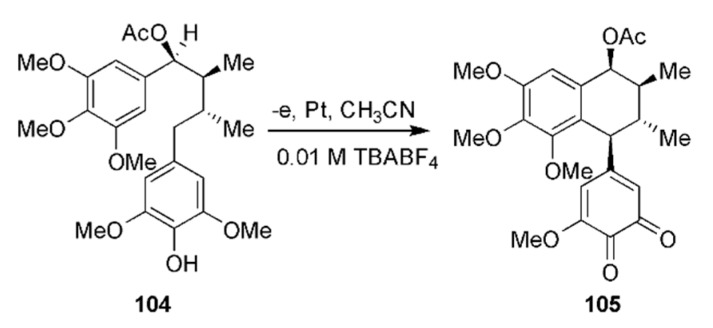
Electrochemical oxidation of an acetoxy lignan to an *o*-quinone.

An acetoxy lignan (**104**) (Scheme 49) has been electrochemically oxidized to an *o*-quinone (**105**) at a constant anode potential of 0.725 V using a platinum gauze working electrode, a platinum wire counter electrode and an Ag/Ag^+^ reference electrode. The electrolyte was a solution of 0.01 M tetrabutylammonium tetraboroflourate in acetonitrile, with solid potassium carbonate as a buffer. The product was not isolated, but further reduced to a catechol, which was isolated and characterized [160].

**Scheme 50:**
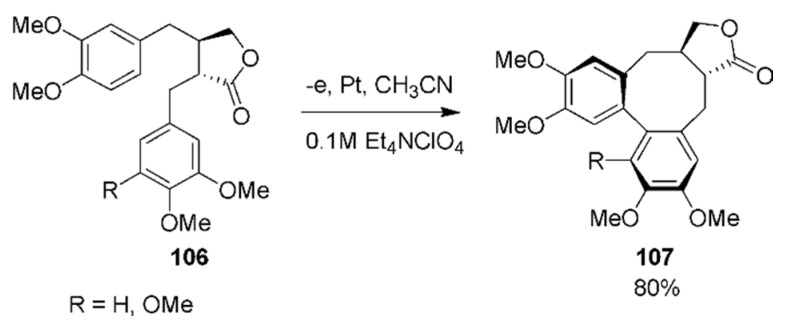
Synthesis of dibenzocyclooctadienes by electrochemical oxidation of butyrolactone lignans.

Dibenzocyclooctadienes have been synthesized by electrochemical oxidation of butyrolactone lignans (**106**) (Scheme 50). A platinum working electrode, a platinum counter electrode, a Ag/AgBr, Et_4_NBr reference electrode, and a 0.1 M Et_4_NClO_4_-CH_3_CN supporting electrolyte was used. The products (**107**) were obtained in high yields, more than 80% in both cases [161].

**Figure 1:**
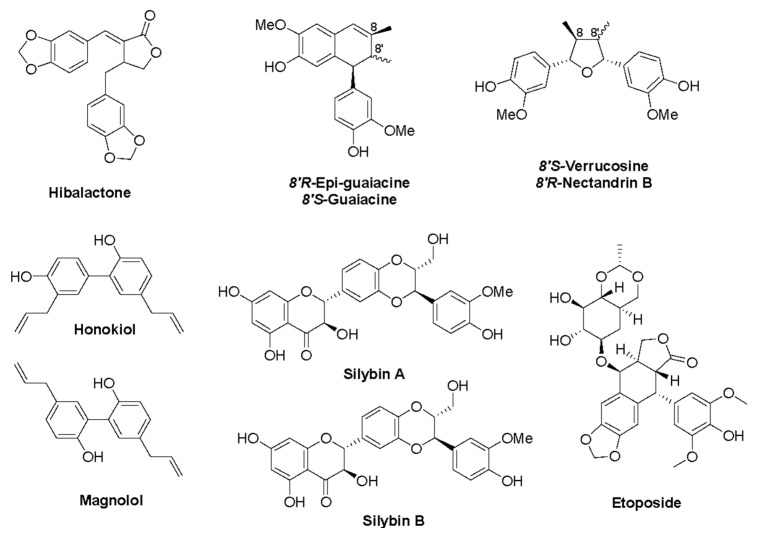
The structures of the lignans hibalactone, *epi*-guaiacin, guaiacin, verrucosin, nectandrin B, honokiol and magnolol, the glycoside etoposide, and the flavonolignan silybin.

The lignan hibalactone has been electrochemically oxidized using voltammetry with a three-electrode system, consisting of a glassy carbon working electrode, a platinum counter electrode, and a Ag/AgCl counter electrode. The lignan showed a single quasi-reversible electro-oxidation, with the alkene bonded to the lactone ring being the most probable site of oxidation [181]. The lignans *epi*-guaiacin, guaiacin, verrucosin, and nectandrin B have been isolated from *Iryanthera juruensis* fruits and electrochemically oxidized during an investigation of their antioxidative properties (Figure 1). A glassy carbon working electrode, a carbon counter electrode, and a Ag/AgCl reference electrode were used [182]. Honokiol and magnolol were electrochemically oxidized using an acetylene black nanoparticle-modified glassy carbon electrode as a working electrode, with a platinum wire counter electrode and a saturated calomel reference electrode. The results indicated that the oxidation of honokiol was reversible and involved two electrons. The oxidation of magnolol was irreversible [183]. 

Foodstuff and plant extracts containing phenolic compounds including lignans have been electrochemically oxidized during the determination of the phenolic content [184,185,186,187] and the antioxidant capacity [188,189]. The drug etoposide, which is a semisynthetic epipodophyllotoxin glycoside, has been oxidized electrochemically using a carbon paste working electrode, a Ag/AgCl/3 M KCl reference electrode, and a platinum wire counter electrode, with a supporting electrolyte of varying pH. The oxidation involves the transfer of two electrons and proceeds in one voltammetric oxidation step at a pH < 4.0 and two voltammetric oxidation steps at a pH > 4.0. In the first and reversible oxidation step, one electron is transferred, resulting in a stable radical. The product in the second oxidation step is an unstable cation, which rapidly converts into the *o*-quinone [190]. The flavonolignan silybin and its derivatives has been electrochemically oxidized using various methods [191,192,193,194,195]. 

### 4.3. Photooxidations

**Scheme 51:**
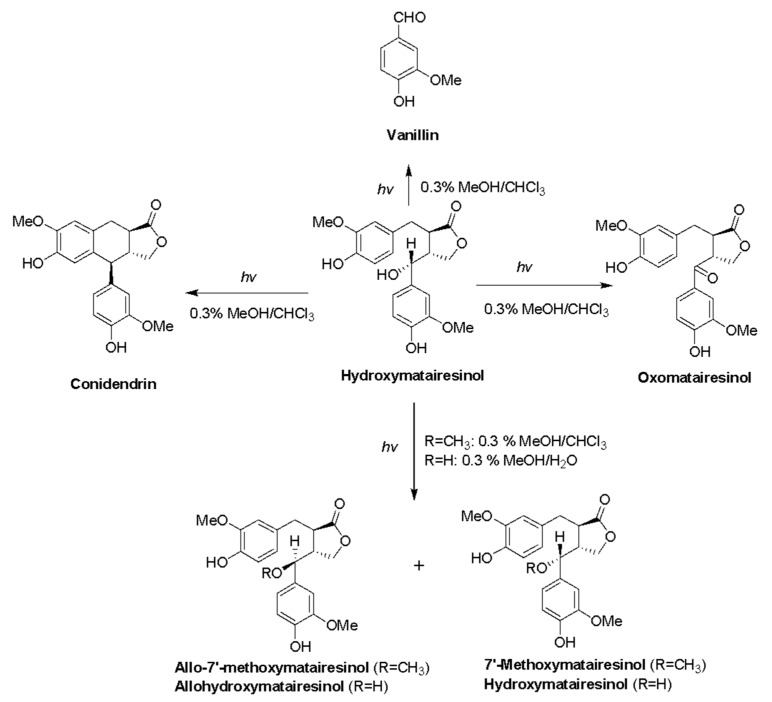
The photooxidation reactions of hydroxymatairesinol.

The lignans hydroxymatairesinol, allohydroxymatairesinol, α-conidendrin, and oxo-matariresinol have been used as substrates for light-irradiation experiments in different solvents. The products of light-irradiation of hydroxymatairesinol that were either isolated or detected were allohydroxymatairesinol, oxomatairesinol, α-conidendrin, allo-7′-methoxymatairesinol, 7′-methoxymatairesinol, and vanillin (Scheme 51). The irradiation of allo-hydroxymatairesinol formed the reaction products hydroxymatairesinol, oxomatairesinol, α-conidendrin, allo-7′-methoxymatairesinol, 7′-methoxymatairesinol, and vanillin. Oxomatairesinol was formed from the irradiation of hydroxymatairesinol, and vanillin from the irradiation of oxomatairesinol [162].

## 5. Conclusions

Lignans are oxidatively transformed in a number of processes and conditions. In nature, oxidations are mostly related to biosynthetic pathways, or processes where lignans act as primary antioxidant scavenging free radicals. When it comes to targeted oxidative transformations or studies between lignans and oxidants, the reports in the literature can be divided into non-metal mediated and metal mediated oxidations. The oxidative transformations include the oxidation of alcohols, both primary and benzylic, or benzylic functionalization by halogenation, hydroxylation, dehydrogenation, or by ring closing reactions. Oxidative Ar-Ar couplings forming cyclooctadienes have been extensively reported and can be quite selectively accomplished with several reagents in high yields. α-Hydroxylations and epoxidations have also been reported, although to a much lesser extent and these reactions may warrant further investigations. Radical mediated oxidations often result in polymerization or radical addition reactions giving a wide range of products with poor selectivity, especially in the presence of free phenolic groups. Although there are over 200 papers reporting the oxidative transformations of lignans, we can conclude that there is still room for further investigations, especially concerning the selectivity. In addition, electrochemical oxidations and photooxidations are rather unexplored and could be a future area of research. 

## Figures and Tables

**Table 1:** Various methods for oxidative transformations of lignans.

**Non-Metal Ox.**		**Oxidative Transformation**	**Yields**	**Ref.**
DDQ		Benzylic hydride abstraction and benzylic ring closures, Ar-Ar-coupling to dibenzocyclooctadiene, nucleophilic attack, alcohol oxidation, aromatization	~10–40%	[18,19,20,21,22,23,24,25,26,27,28,29,30,31,32,33,34,35,36,37]
*m*CPBA		Epoxidation, Baeyer-Villiger oxidation, oxidation of ethoxy-THF-lignans to lactones	~70–90%	[29,38,39,40,41,42]
peroxyl radical (ROO^.^)		Antioxidative radical scavenging to phenoxyl radicals and further radical couplings	No data	[43,44,45,46]
Azo compounds (AAPH, AIBN, ABTS)		Same as above	No data	[46,47,48,49,50,51,52,53,54,55,56,57,58,59,60,61,62,63]
DPPH		Same as above, radical 5-5 couplings to dimers and oxidation of benzylic alcohol to ketone	No data	[6,51,64,65,66,67]
TEMPO		Oxidation of benzylic alcohol to ketone	60%	[68]
BAIB and PIFA		phenolic hydroxyl oxidation to quinone type structures, Ar-Ar-coupling to dibenzocyclooctadienes, nucleophilic attack at *ipso*- or benzylic position	~10–80%	[69,70,71,72,73,74]
IBX		Aromatic demethylation, benzylic alcohol oxidation to carbonyl	~25–95%	[75,76,77,78,79]
Dess-Martin Periodinane (DMP)		Benzylic alcohol oxidation to carbonyl, 9,9′-diol oxidation to lactols	>90%	[80,81,82,83,84,85,86,87,88,89]
NaIO_4_		Oxidation of guaiacyl or syringyl groups to demethylated *o*-quinones, Lemieux–Johnson oxidation	~50–95%	[90,91,92,93,94,95,96,97,98,99,100]
*N*-Bromosuccinimide (NBS)		Brominations (arylic or benzylic), benzylic CH_2_ to ketone, benzylic ring closure and aromatization	75–90%	[35,101,102,103,104,105,106,107]
Dimethyldioxirane (DMDO)		Oxidative ring opening of furan rings	~80%	[108]
Nitrobenzene		Oxidative degradation to vanillin and vanillic acid	80–100%	[109,110,111]
**Metal-Mediated Ox.**		**Oxidative Transformation**	**Yields**	**Ref.**
Cr-(VI) (CrO_3_, PCC, PDC)		Oxidation of benzylic alcohols to ketones, primary alcohols into lactones and carboxylic acid	~60–95%	[87,103,105,112,113,114,115,116,117,118,119,120]
Pd, Au		Oxidation of benzylic alcohols in presence of free phenolic into (mainly) ketone	No data	[121,122,123,124,125,126]
MoOPH		α-Hydroxylation	~25–95%	[113,127,128,129,130,131]
MoCl_5_		Ar-Ar-coupling to dibenzocyclooctadienes	~50–90%	[132,133]
VoF_3_; V_2_O_5_; Tl_2_O_3_; RuO_5_		Ar-Ar-coupling to dibenzocyclooctadienes, benzylic ring closure	~50–100%	[134,135,136,137,138,139,140,141,142,143,144,145,146,147]
MTO (catalyst)		Aromatic demethylation and quinone formation and simultaneous benzylic alcohol oxidation to ketones, benzylic cleavage, benzylic hydroxylation, oxidation of benzylic alcohol to ketone	~40–100%	[148,149,150]
Pb(OAc)_4_		Benzylic acetoxylation	~30–70%	[151]
CeCl_3_		α-Hydroxylation	71%	[152]
**Other Oxidations**		**Oxidative Transformation**	**Yields**	**Ref.**
Enzymatic ox.	peroxidase	Benzylic ring closure	37–99%	[153,154,155]
	HRP	Radical 4-*O*-5 coupling	3.6%	[156]
	SDH	Oxidation of diol to lactone	No data	[157]
	P450/CPR1	Arylic hydroxylation and rearrangement	No data	[158]
	CYP	Benzylic hydroxylation	90%	[159]
Electrochemical ox.		Demethylation, quinone formation, and benzylic ring closure	No data	[160]
		Ar-Ar-coupling to dibenzocyclooctadiene	>80%	[161]
Photooxidation		Benzylic: cleavage/alcohol oxidation to ketone/ring closure/nucleophilic attack	No data	[162]

**Table 2:** Results of nitrobenzene-mediated oxidative degradation of lignans.

Lignan	Vanillin %	Vanillic Acid %	Conversion %
Pinoresinol	31	9	81
Lariciresinol	63	5	100
Olivil	83	3	100
Matairesinol	15	2	100
Conidendrin	1	-	-
Isoolivil	3	-	-
